# Characterization of a Type VI Secretion System *vgrG2* Gene in the Pathogenicity of *Burkholderia thailandensis* BPM

**DOI:** 10.3389/fmicb.2021.811343

**Published:** 2022-01-05

**Authors:** Jin Li, Wei-wei Hu, Guo-xin Qu, Xiao-rong Li, Yi Xiang, Peng Jiang, Jiang-qiao Luo, Wen-huan He, Yu-jia Jin, Qiong Shi

**Affiliations:** ^1^M.O.E. Key Laboratory of Laboratory Medicine Diagnostics, Department of Laboratory Medicine, Chongqing Medical University, Chongqing, China; ^2^Department of Respiratory and Critical Care Medicine, The First Affiliated Hospital of Chongqing Medical University, Chongqing, China; ^3^Department of Orthopedic Surgery, The First Affiliated Hospital of Hainan Medical University, Hainan, China

**Keywords:** BPM, pathogenicity, virulence factor, T6SS, *vgrG2* gene

## Abstract

*Burkholderia thailandensis* is a clinically underestimated conditional pathogen in the genus *Burkholderia*, the pathogenicity of the infection caused by *B. thailandensis* remains poorly understood. According to previous studies, Type-VI secretion system (T6SS) is a protein secreting device widely existing in Gram-negative bacilli. Valine-glycine repeat protein G (VgrG) is not only an important component of T6SS, but also a virulence factor of many Gram-negative bacilli. In one of our previous studies, a unique T6SS *vgrG* gene (*vgrG2* gene) was present in a virulent *B. thailandensis* strain BPM (BPM), but not in the relatively avirulent *B. thailandensis* strain E264 (E264). Meanwhile, transcriptome analysis of BPM and E264 showed that the *vgrG2* gene was strongly expressed in BPM, but not in E264. Therefore, we identified the function of the *vgrG2* gene by constructing the mutant and complemented strains in this study. *In vitro*, the *vgrG2* gene was observed to be involved in the interactions with host cells. The animal model experiment showed that the deletion of *vgrG2* gene significantly led to the decrease in the lethality of BPM and impaired its ability to trigger host immune response. In conclusion, our study provides a new perspective for studying the pathogenicity of *B. thailandensis* and lays the foundation for discovering the potential T6SS effectors.

## Introduction

*Burkholderia thailandensis* is a clinically underestimated conditional pathogen in the genus *Burkholderia.* It is very similar to *Burkholderia pseudomallei* in terms of colony morphology, immunogenicity and antimicrobial susceptibility (Brett et al., 1998; Ngamdee et al., 2015; Garcia, 2017). To date, only a few studies have described the isolation of *B. thailandensis* from invasive human infections (Lertpatanasuwan et al., 1999; Glass et al., 2006; Ginther et al., 2015; Gee et al., 2018), and the pathogenic mechanism of the infections caused by *B. thailandensis* remains poorly understood. Therefore, it is necessary to study the pathogenesis of *B. thailandensis* to more effectively prevent infections caused by *B. thailandensis*. In one of our previous studies, the virulent *B. thailandensis* strain BPM was isolated from a blood and sputum specimen of a 76-year-old man with sepsis who died in China (Chang et al., 2017). The biological and biochemical characteristics of BPM are very similar to those of *B. pseudomallei*, and the clinical symptoms and imaging findings of patients infected with this strain are consistent with acute melioidosis, manifesting as acute high fever, productive cough with white sputum and breathing difficulty (Wiersinga et al., 2012; Tang et al., 2018; Gassiep et al., 2020). However, biochemical identification test results showed that BPM was positive for arabinose assimilation, which is consistent with the biochemical characteristics of *B. thailandensis*. To confirm the biochemical identification results, 16S rRNA gene sequence and whole-genome sequencing analyses were performed. The results showed that the sequence of the 16S rRNA gene was 100% consistent with that of the *B. thailandensis* E264 (E264) 16S rRNA gene (GenBank No. CP008785.1, CP008786.1) and that the sequence obtained by whole-genome sequencing was more than 96% homologous to the genome sequence of E264 (GenBank No. CP000085.1, CP000086.1). Finally, the BPM strain was identified as *B. thailandensis* based on the NT nucleic acid sequence database, and the genome sequences of BPM have been deposited in the GenBank database under accession numbers CP050020-CP050021. In one of our previous studies, we compared the virulence of BPM and E264 in BALB/c mice, and the results indicated that the virulence of BPM was significantly higher than that of E264, which confirmed that BPM is a virulent pathogen (Chang et al., 2017). Additionally, an integrated type VI secretion system (T6SS) gene cluster was found in BPM by bioinformatic analysis. However, the pathogenicity of the T6SS involved in BPM infection is poorly understood.

The T6SS is an important virulence factor that plays a key role in microbial competition and bacterial infection (Fridman et al., 2020). It can deliver toxic effectors to bacterial and eukaryotic neighbors and plays an important role in both bacterial competition and virulence (Hsieh et al., 2019). Valine-glycine repeat protein G (VgrG) has been reported to be an important component of the functional T6SS of *B. pseudomallei* and is involved in the occurrence of acute melioidosis (Schwarz et al., 2014). However, the functions of the T6SS *vgrG* gene in the development of *B. thailandensis* infections remain unknown. In one of our previous studies, a unique T6SS *vgrG* gene (*vgrG2* gene) was present in a virulent BPM, but not in the relatively avirulent E264. Meanwhile, transcriptome analysis of BPM and E264 showed that the *vgrG2* gene was strongly expressed in BPM, but not in E264. However, the function of *vgrG2* gene remains unknown. Therefore, this study investigated the function of the putative T6SS *vgrG2* gene in BPM by knocking out the *vgrG2* gene and producing a *vgrG2* gene complementation construct. The mutant and complemented strains will be used to determine the function of the T6SS *vgrG2* gene by investigating the changes in the virulence of BPM. Altogether, this study aimed to lay a foundation for discovering potential T6SS effectors of *B. thailandensis* and provide a new perspective on the study of host cell signal transduction and immune defense mechanisms.

## Materials and Methods

### Bacterial Strains and Growth Conditions

All bacterial strains used in this study are listed in Table 1. All strains were cultured on sheep blood agar plates (Thermo, United States) at 37°C for 16–20 h, and isolated colonies were inoculated into 10 mL Luria-Bertani (LB) broth (Solarbio, China), which was then stirred at 37°C for 12 h. The working cultures were prepared by transferring 100 μL of a 12 h culture to 10 mL LB broth (1:100 dilution), which was then allowed to stand at 37°C for 8 h. The stationary-phase bacteria were diluted to 10^6^∼10^8^ colony-forming units (CFU)/mL in LB broth, and phenotypic characteristics were evaluated (Jiang et al., 2016). These final suspensions were plated onto LB agar to accurately determine the number of CFUs per milliliter.

**TABLE 1:** Bacterial strains used in this study.

Strain	Description	Source or reference
BPM	A hypervirulent strain isolated from a deceased patient with *B. thailandensis* infection, TC^S^, Cms^S^	Laboratory collection
BE264	An environmental strain from the American Type Culture Collection, TC^S^, Cm^S^	United States (ATCC 700388)
Δ*vgrG2*	Mutant with BPMhun02934 gene deleted in BPM, TC^S^, Cm^S^	This study
Δ*vgrG2/*p*vgrG2*	Mutant Δ*vgrG2* complemented with gene *vgrG2*, TC^S^, Cm^S^	This study

### Transcriptomic Analysis

The total RNA of BPM and E264 was extracted with TRIzol reagent (Invitrogen, United States). The quantity and purity of the extracted RNA were assessed using a NanoDrop ND-1000 spectrophotometer (Thermo, United States). RNA-seq libraries were created using the Illumina TruSeq Stranded mRNA Library Prep Kit (Illumina, Inc., United States) according to the manufacturer’s protocol. Sequencing was performed at Shenzhen Hai-yi Biotechnology Co., Ltd. using an Illumina MiSeq System benchtop sequencing instrument (read length: 75 bp, read type: paired end) (Zhu et al., 2016). The raw sequence data were filtered by removing reads containing adapters, reads containing poly N sequences, and low-quality reads. The clean reads were aligned to the genomes of BPM and E264 by using Bowtie2-2.2.3 (Kovacs-Simon et al., 2019). DESeq was used to identify differentially expressed genes (Zhu et al., 2016).

### Construction of Mutant and Complemented Strains

To test the role of the T6SS *vgrG2* gene in the pathogenesis of *B. thailandensi*s and its contribution to the development of *B. thailandensi*s infection, knockout mutants of a key component (*vgrG2*) of the T6SS were constructed by double crossover recombination through allelic replacement of the suicide plasmid pLP12cm as described previously (Luo et al., 2015). The knockout mutant was designated Δ*vgrG2*. The *vgrG2* gene was amplified from the *B. thailandensi*s BPM genome and then ligated into plasmid pTac-tetM to construct the complementation expression plasmid pTac-tetM-*vgrG2*. Finally, the complementation plasmids were transferred into mutants to generate complemented strains (Δ*vgrG2/*p*vgrG2*). All mutant and complemented strains were verified using PCR (Supplementary Figure 1) and DNA sequencing (data not shown).

### Growth Characteristics and Antimicrobial Susceptibility Testing

The strain was cultured on sheep blood agar at 37°C for 18–20 h and then transferred to LB broth for shaking culture at 180 rpm at 37°C. The growth characteristics of the BPM, mutant, and complemented strains were determined via optical density measurements (Eppendorf BioPhotometer, Germany) performed at 600 nm (OD600), and colony formation units (CFUs) were counted over a 24-h period as described previously (Zhu et al., 2020). Then, the antimicrobial susceptibilities of the BPM, mutant, and complemented strains were initially tested with a Vitek-2 Compact automatic microbiological assay system (BioMérieux, French). The experimental methods were performed according to the guidelines of the Clinical and Laboratory Standards Institute (CLSI) for *P. aeruginosa* (Bobenchik et al., 2017). Fresh bacterial colonies extracted directly from sheep blood agar were incubated at 37°C for 18–24 h and then resuspended in sterile saline to obtain a suspension of 0.5 McFarland turbidity. *E. coli* ATCC 25922 and *P. aeruginosa* ATCC 2785 were used as quality controls. The antimicrobial susceptibility testing results were explained in accordance with the CLSI M45 guidelines for *B. pseudomallei*. Each assay was performed three times.

### Animal Model Experiments

All animal experiments were approved by the research board of the Ethics Committee of the Third Military Medical University under permit number AMUMEC-20201085. To determine the 50% lethal dose (LD_50_), five-week-old, pathogen-free, female BALB/c mice were obtained from Daping Hospital Animal Center. Ten BALB/c mice were used as a sample population for the survival rate of BALB/c mice infected with BPM, mutant, and complemented strains. Phosphate-buffered saline (PBS) was used as negative control. Ten BALB/c mice were selected for each bacterial concentration to determine the LD_50_. Two-fold serial dilution of the bacteria was performed from a starting concentration of 8 × 10^7^CFU/mL to 5 × 10^6^CFU/mL, and BALB/c mice were infected intravenously with 0.1 mL of each concentration. Symptoms and mortality rates were observed for seven days. The exact inoculation dose was confirmed on LB agar, and the LD_50_ was calculated as described by Barnes (Barnes and Ketheesan, 2005).

### Histopathological Studies

To examine the differences in the pathological changes caused by the tested strains, livers and lungs were collected from BALB/C mice infected with the BPM, mutant and complemented strains at designated times (4, 8, 12, and 16 h post infection). Tissue samples were fixed in 10% buffered formalin. Paraffin-embedded tissue sections were stained with hematoxylin and eosin according to the standard protocol and examined by light microscopy (Zhao et al., 2011).

### Systemic Measurement of Inflammatory Cytokines

To assess the function of the *vgrG2* gene in inflammation, serum samples (infected with BPM, mutant, and complemented strains) were collected, and the levels of IL-1β, IL-6 and TNF-α were measured using Mouse Precoated ELISA kits (Dakewei Biotech Co., Ltd). Each assay was performed three times.

### Whole-Blood Bactericidal Experiments

Human whole-blood samples used in the experiment were taken from 10 healthy individuals. The whole blood bactericidal assay was performed as previously described with minor modifications (Zong et al., 2019). Briefly, a bacterial inoculum of 100 μL (adjusted to 10^6^ CFU/mL) prepared from the mid-log phase was diluted with PBS and added to 900 μL of fresh whole blood contained in 24-well plates (Corning, United States), and the mixtures were incubated at 37°C. After incubation for 3 h, the bacteria were plated onto LB agar and counted. The survival rates of the BPM, mutant, and complemented strains were expressed by using the following formula: (CFU/mL)_t=3h_)/(CFU/mL)_t=0h_) × 100%. Each assay was performed three times.

### Cell Invasion and Survival Assays

The cell invasion assay was similar to that previously performed (Pijuan et al., 2019). RAW264.7 cells were incubated at 37°C with 5% CO_2_ in 24-well plates at a concentration of 5 × 10^5^ cells per well. RAW264.7 cells were grown on DMEM (Gibco GlutaMAX™, United States) containing glucose, glutamine, and 10% fetal bovine serum. *B. thailandensi*s suspensions were added to the cells at an MOI of 10 or 100, followed by centrifugation at 500 g for 5 min and incubation at 37°C with 5% CO_2_ for 1 h to determine invasion. One-hour post infection (hpi), the monolayers were washed twice with PBS and lysed with 0.1% Triton X-100 (Sigma, United States) in PBS, and serial dilutions were plated and incubated at 37°C for 48 h. The invasion percentages of the BPM, mutant, and complemented strains were calculated as follows: (invasion CFU/total inoculum CFU) × 100 (Lewis et al., 2017). To determine intracellular survival after initial invasion, after 1 h, the monolayers were washed twice with PBS and replenished with complete medium containing 250 μg/ml chloromycetin. At 3 h post inoculation, the monolayers were washed twice with PBS and then lysed with 0.1% Triton X-100 in PBS, and serial dilutions were plated and incubated at 37°C for 48 h. The percent survival of the BPM, mutant, and complemented strains was calculated as (survival CFU/invasion CFU) × 100 (Lewis et al., 2017). Each assay was performed three times.

### Cell Counting Kit 8 Assays

The cytotoxicity of the bacteria to RAW264.7 cells was tested by CCK-8 assays. Bacteria in the stationary phase resuspended in fresh medium were added to 96-well plates (Corning, United States) (MOI = 10). RAW264.7 cells were washed with PBS, resuspended in DMEM and plated in 96-well plates at a concentration of 5000 cells/well. Next, CCK-8 assay kit (MCE, China) reagents were added to the wells according to the manufacturer’s instructions. The optical density at 450 nm was measured using a microplate reader (Thermo Fisher Scientific, Varioskan LUX, China) to assess cell viability. Cytotoxicity was expressed according to the following formula: cytotoxicity (%) = (test sample – low control)/(high control – low control) × 100 (Tang et al., 2021). Each assay was performed three times.

### Statistics

Statistical analyses were performed using GraphPad Prism 7 (San Diego, United States). One-way ANOVA with the log-rank test was used to compare BPM to the mutant and complemented strains. We also used Tukey’s multiple comparison test to compare each strain to all other strains. Significant differences between groups are indicated: * (*P* < 0.05), ** (*P* < 0.01) and *** (*P* < 0.001).

## Results

### Transcriptomic Analysis of BPM and E264

**FIGURE 1:**
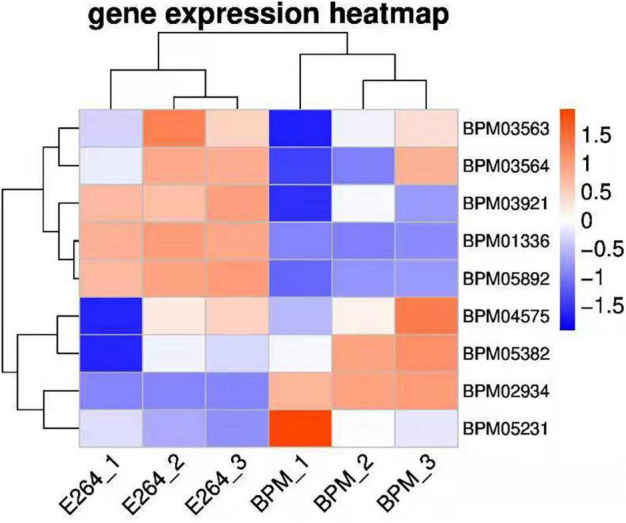
Heatmap of *vgrG* gene expression in BPM and E264. Colors in the heatmap represent gene expression levels among samples. The BPM and E264 results came from three repeated samples.

We sequenced and analyzed the transcriptomes of BPM and E264 and submitted the transcriptome data to the NCBI database to obtain the sequence and annotation information of the transcriptome sequencing assembly (number: GSE147369). Relative to E264, there was no difference in the expression of six BPM homologs of *vgrG* genes (*BPM03563*, *BPM03564*, *BPM03921*, *BPM04575*, *BPM05231*, and *BPM05382*), while the expression of *BPM02934* (*vgrG2*) was upregulated, and the expression of *BPM01336* and *BPM05892* was downregulated (Table 2). The heatmap of *vgrG* gene expression is shown in Figure 1, which indicated that the *vgrG2* gene is a unique virulence factor. The VgrG protein is a needle-like structure of the T6SS and is homologous to the T4 bacteriophage cell-puncturing device, which contributes to the development of acute melioidosis (Schwarz et al., 2014). Therefore, the *vgrG2* gene was selected and subjected to further experiments in our study.

**TABLE 2:** Expression of *vgrG* gene in BPM.

GeneID	E264_expr	BPM_expr	log_2_ Fold	p value	q value	Diff
BPM01336	100.6032	48.7003	−1.267132379	1.43E-52	3.02E-51	Down
BPM02934	0	44.2911	11.34104488	9.68E-22	6.33E-21	Up
BPM03563	2.0684	1.492	−0.709531306	0.079886227	0.115328157	-
BPM03564	2.9132	2.1304	−0.691301852	0.042559877	0.064857862	-
BPM03921	17.5007	13.6585	−0.580339578	0.000169016	0.000357747	-
BPM04575	0.7314	1.0029	0.186528779	0.709326414	0.761237862	-
BPM05231	4.877	6.4538	0.204416147	0.386627009	0.460374412	-
BPM05382	1.0218	1.5411	0.344760554	0.39838535	0.472371201	-
BPM05892	91.3012	45.9427	−1.221455825	3.57E−32	3.70E−31	Down

*The first column is the gene ID; the second and third column are the standardized expressions of E264 and BPM; the fourth column is the ratio of normalized expression (BPM/E264, log_2_ fold change in transcriptome); the fifth column is the corrected p-value; the sixth column is the corrected q-value; the seventh column indicates genetic differences, Up indicates up-regulation, Down indicates down-regulation, noDEG indicates no difference.*

### Growth Characteristics and Antimicrobial Susceptibility Analysis of BPM, Mutant and Complemented Strains

Growth rates were plotted according to the measured OD_600_ values and CFUs as described previously (Zong et al., 2019). No growth rate difference was found when the mutant and complemented strains were compared with BPM (Supplementary Figure 2). The antimicrobial susceptibility results of the mutant and complemented strains related to six antibiotics were consistent with those of BPM, as shown in Supplementary Table 1. All of these strains were sensitive to amoxicillin/clavulanate, ceftazidime, imipenem, tetracycline, doxycycline, and trimethoprim/sulfamethoxazole.

### Survival Rate and LD_50_ of BALB/c Mice Infected With BPM, Mutant and Complemented Strains

**FIGURE 2:**
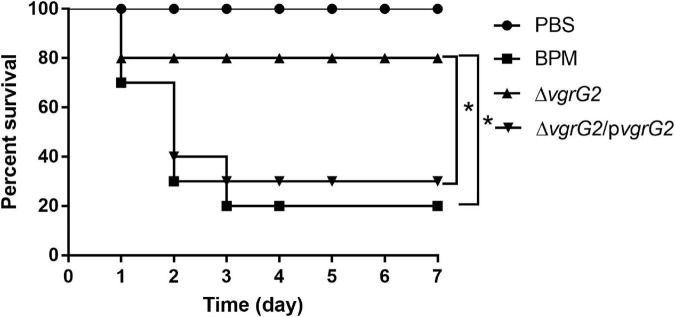
Survival rate of BALB/c mice infected with BPM, mutant and complemented strains. The mortality of BALB/c mice after the intraperitoneal injection of all strains was observed over 7 days. Data points represent the percentage of BALB/c mouse survival in each group (*n* = 10 mice per strain and 1 × 10^7^CFU per mouse). After infection for 7 days, the survival rate of BALB/c mice infected with PBS and Δ*vgrG* was significantly lower than that of BALB/c mice infected with BPM *(*P* < 0.05).

To determine whether the deletion of the *vgrG2* gene impairs the virulence of BPM, the survival rate and LD_50_ of BALB/c mice infected with the BPM, mutant and complemented strains were compared. After infection for 7 days, the survival rate of BALB/c mice infected with BPM and Δ*vgrG2/*p*vgrG2* was significantly lower than that of BALB/c mice infected with Δ*vgrG2* (Figure 2, *P < 0.05). The LD_50_ results showed that the LD_50_ of Δ*vgrG2* was 1.61 × 10^7^ CFU (Table 3), which indicates low virulence. In contrast, BPM and Δ*vgrG2/*p*vgrG2* showed relatively high virulence, with LD_50_ values of 8.35 × 10^6^ CFU and 8.87 × 10^6^ CFU, respectively (Table 3). Their phenotypic characteristics indicated that the *vgrG2* gene was involved in the virulence of BPM in BALB/c mice.

**TABLE 3:** LD_50_ of BPM, mutant and complemented strains in BALB/c mice.

	Number of Deaths/Total			Mortality (%)		
Dose of Challenge CFU	WT	Δ*vgrG2*	Δ*vgrG2*/p*vgrG2*	WT	Δ*vgrG2*	Δ*vgrG2/pvgrG2*
8 × 10^7^	10/10	10/10	10/10	100%	100%	100%
4 × 10^7^	10/10	10/10	10/10	100%	100%	100%
2 × 10^7^	10/10	6/10	10/10	100%	60%	100%
1 × 10^7^	8/10	2/10	7/10	80%	20%	70%
5 × 10^6^	0/10	0/10	0/10	0%	0%	0%
LD_50_				8.35 × 10^6^	1.61 × 10^7^	8.87 × 10^6^

### Pathological Characteristics

**FIGURE 3:**
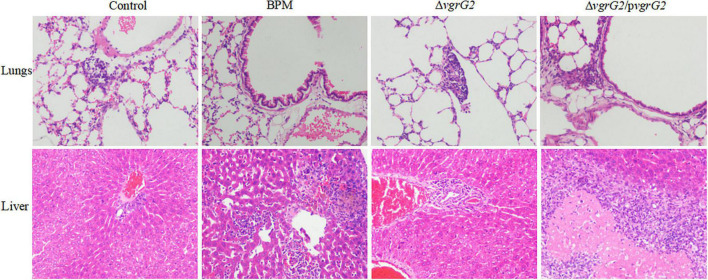
Pathological characterization of lungs and liver tissues of BALB/c mice infected with BPM, mutant and complemented strains. Lungs and liver tissues of BALB/c mice infected with BPM, mutant, complemented strains and PBS (control) were prepared for light microscopy analysis and examined for differences in pathological changes (hematoxylin and eosin staining; original magnification × 200).

During the first 8 h after infection, BALB/C mice infected with PBS and the three indicator strains showed no significant histopathological changes in the liver or lungs (data not shown). At 24 h after infection, different histopathological changes were observed in the lungs and livers of BALB/c mice infected with PBS and the three indicated strains. As shown in Figure 3, after BALB/C mice were infected with the three indicated strains, a small number of inflammatory cells infiltrated the lung tissue, central vein and convergence area, and liver tissue necrosis and partial destruction of the liver cell structure were observed.

### Deletion of *vgrG2* Decreases the Production of Inflammatory Cytokines

**FIGURE 4:**
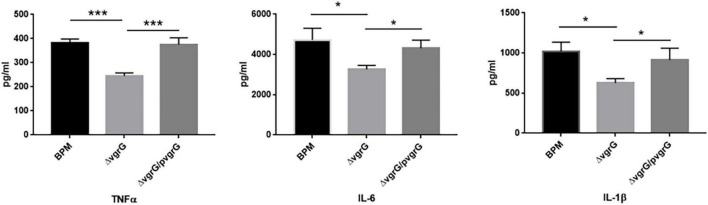
Serum levels of cytokines in BLAB/c mice 16 h after infection with BPM, mutant and complemented strains. Serum IL-1β, IL-6, and TNF-α levels in BALB/c mice 16 h after infection with the BPM, mutant and complement strains. All data are from three independent experiments. Significant differences between groups are indicated: *(*P* < 0.05) and ***(*P* < 0.001).

To determine whether the *vgrG2* gene is involved in the expression of proinflammatory cytokines, serum samples were collected from intravenously infected BALB/c mice for analysis of proinflammatory cytokines. Proinflammatory cytokines were detected in BALB/c mice 8 h after infection with the BPM, mutant and complemented strains. As shown in Figure 4, the production of TNF-α triggered by BPM and Δ*vgrG2/*p*vgrG2* was significantly higher than that triggered by Δ*vgrG2* (*** *P* < 0.001). The levels of IL-1β and IL-6 induced by BPM and Δ*vgrG2/*p*vgrG2* were relatively higher than those induced by Δ*vgrG2* (* *P* < 0.05).

### Survival Rates of BPM, Mutant and Complemented Strains in Whole Blood

**FIGURE 5:**
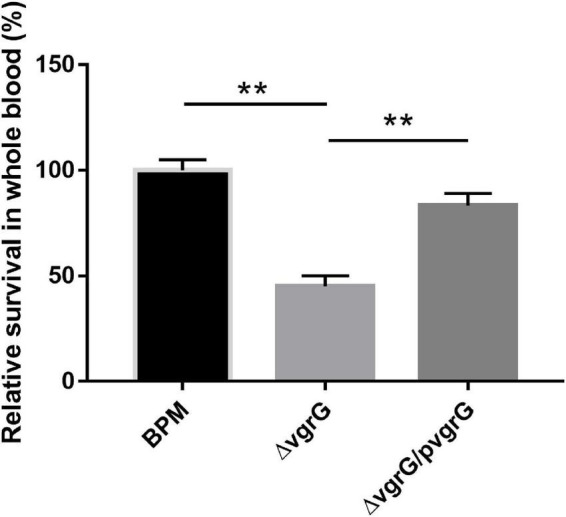
Whole-blood bactericidal experiments of BPM, mutant and complemented strains. Survival rates of the BPM, mutant and complemented strains in human whole blood. The survival rates are expressed relative to those of BPM (100%). Means and SDs of three independent experiments performed in triplicate were calculated. Significant differences between groups are indicated: **(*P* < 0.01).

To evaluate the function of the *vgrG2* gene in the evasion of innate immune responses, we measured the survival rates of the BPM, mutant and complemented strains in whole blood collected from healthy individuals. The experimental results showed that the survival rate of Δ*vgrG2* was significantly lower than those of BPM and Δ*vgrG2/*p*vgrG2* (Figure 5, ** *P* < 0.01).

### Interaction Between Bacteria and RAW264.7 Cells

**FIGURE 6:**
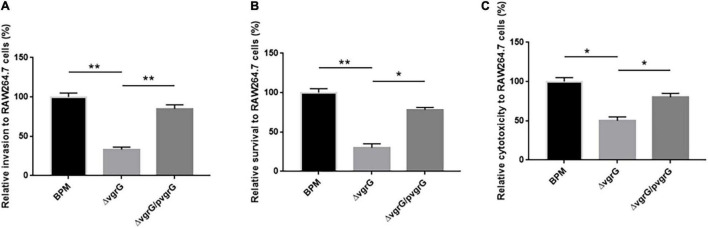
Interaction between bacteria and RAW264.7 cells. **(A–C)** indicate the invasion ability, intracellular survival ability, and the cytotoxicity, respectively, of the BPM, mutant and complemented strains in RAW264.7 cells. Rates of invasion, survival and cytotoxicity are expressed relative to those of BPM (100%). The means and SDs of three independent experiments in conducted triplicate were calculated. Significant differences between groups are indicated: *(*P* < 0.05) and **(*P* < 0.01).

To further investigate whether the *vgrG2* gene completely or partially impairs T6SS activity, we compared the cell invasion, intracellular survival and cytotoxicity of the mutant and complemented strains with those of BPM. The results showed that the cell invasion, intracellular survival and cytotoxicity of Δ*vgrG2* were significantly lower than those of the BPM and Δ*vgrG2/*p*vgrG2* (Figure 6), and no significant difference in cell invasion, intracellular survival or cytotoxicity was found between the BPM and Δ*vgrG2/*p*vgrG2* (Figure 6), suggesting that the deletion of *vgrG2* affected the cell invasion, intracellular survival and cytotoxicity of BPM. Since *vgrG* gene has been reported as a virulence factor of functional T6SS in *B. pseudomallei*, it can be concluded that the deletion of *vgrG2* may impair the overall activity of T6SS by affecting the assembly of T6SS in BPM.

## Discussion

T6SS widely occurs in approximately 25% of all sequenced Gram-negative bacteria, including members of the genera *Vibrio*, *Pseudomonas*, *Burkholderia*, *Serratia*, *Edwardsiella*, and *Enterobacter* (Chieng et al., 2015; Gerc et al., 2015; Wood et al., 2019; Crisan and Hammer, 2020). T6SS plays an important role in pathogenicity, competition, proliferation, and cooperation (Chen et al., 2015). The T6SS is structurally, functionally, and evolutionarily related to contractile injection systems (CISs), a broad family of machines with a spring-like mechanism for delivering macromolecules into target cells (Douzi et al., 2018; Navarro-Garcia et al., 2019). A series of *Burkholderia* virulence factors, including secreted toxins, adhesins, iron acquisition systems, T6SS, and BLF1, have been reported (Bernhards et al., 2017; Lennings et al., 2018; Rust et al., 2018). Recent studies have indicated that T6SS plays an important role in the competition and pathogenicity of *Burkholderia* (Chieng et al., 2015). Our previous studies have shown that the clinical symptoms and imaging findings of patients with BPM infection are consistent with those of acute melioidosis (Chang et al., 2017). Therefore, we believe that hypervirulent *B. thailandensis* may pose a significant threat to human public health, and it is important to study the potential virulence-associated genes involved in BPM. In one of our previous studies, nine *vgrG* genes were found in the BPM genome. Further sequence analysis showed that only the *vgrG2* gene was specific to BPM. Additionally, transcriptome analysis of BPM and E264 showed that the *vgrG2* gene was strongly expressed in BPM but not in E264. Therefore, we hypothesized that the *vgrG2* gene is involved in the function of the T6SS. To test our hypothesis and describe the role of the *vgrG2* gene in BPM, a series of experiments were carried out.

To study the effect of the *vgrG2* gene on the pathogenicity of BPM, knockout mutants and complemented strains of the *vgrG2* gene were developed from BPM, and there were no significant differences in growth characteristics and antimicrobial sensitivity between them. In addition, the survival rate and LD_50_ of BALB/c mice infected with these strains were compared. In animal model experiments, we found that the survival rate and LD_50_ of BALB/c mice infected with Δ*vgrG2* were higher than those of BALB/c mice infected with BPM, which demonstrated that the deletion of the *vgrG2* gene significantly weakened the virulence of BPM. It has been reported that the T6SS can activate the inflammasome and cause inflammation (Aubert et al., 2016; Ratner et al., 2017; Loeven et al., 2021). To further study the function of the *vgrG2* gene in BPM pathogenesis, the serum levels of TNF-a, IL-1β and IL-6 in BALB/c mice were detected. We found that the level of IL-1β was significantly reduced after the deletion of the *vgrG2* gene. These findings indicated that the *vgrG2* gene of the T6SS in BPM plays an important role in the pathogenicity of BPM, which was consistent with previous reports (Aubert et al., 2016). Unexpectedly, no significant differences in inflammatory cell infiltration were observed in the lungs and livers of the mice after stimulation with these different strains, particularly at 8 h after infection. The results of animal model experiments showed that the deletion of the *vgrG2* gene led to the elimination of BPM lethality and a decrease in serum cytokine levels in BALB/c mouse serum.

The evasion of innate immune responses was reported to be very important for the survival and pathogenicity of *P. aeruginosa* and *B. pseudomallei* (Gong et al., 2011; Alonso et al., 2020). In this study, our goal was to assess whether the BPM and complemented strains differ from Δ*vgrG2* in terms of their virulence and lethality. We accomplished this by measuring the survival rates of the indicated strains in whole blood. The experimental results indicated that the *vgrG2* gene may participate in the immune evasion of BPM and play a key role in the evasion of innate immune responses in whole blood. Bacterial adherence to and interaction with RAW264.7 cells are prerequisites for the induction of bacterial infection (Bruballa et al., 2020). We observed the invasion and survival abilities of the BPM, mutant and complemented strains and compared them to the abilities of RAW264.7 cells. We observed some differences when we used an MOI of 10 in our studies. Similarly, only the *vgrG2* gene affected the adherence and invasion abilities of BPM, which was consistent with the above results. In addition, previous studies have reported that the *vgrG* gene in *E. coli* and *B. pseudomallei* induced cell toxicity (Hopf et al., 2014; Cianfanelli et al., 2016). Therefore, the cytotoxicity of the BPM, mutant and complemented strains was compared, and the results were consistent with the results obtained from the examination of whole blood killing, adherence and invasion. It could be concluded that the *vgrG2* gene located within the T6SS plays a role in BPM pathogenicity, which is consistent with the hypothesis that the *vgrG2* gene is functional.

In the current study, we were unable to establish a correlation, and the virulence phenotypes of the BPM, mutant and complemented strains were similar *in vitro*, although differences in mortality were observed in the *in vivo* intravenous model of infection. Due to the wide variability of *Burkholderia* virulence properties, we strongly recommend that the selection of the tissue culture cells used in *in vitro* studies should be directly related to the cells found in the organ to which the dose will be delivered *in vivo* (Rao et al., 2020). Therefore, our study attempted to standardize the cell types used for *in vitro* and *in vivo* studies to provide a more meaningful comparison.

In conclusion, our study showed that only the *vgrG2* gene was involved in the whole blood killing of BPM, which promoted the adhesion and invasion of BPM to host cells and enhanced its pathogenicity in the host. Further studies could focus on exploring potential T6SS effectors to facilitate the development of effective antimicrobial agents for the treatment of *B. thailandensis* infection.

## Data Availability Statement

The datasets presented in this study can be found in online repositories. The names of the repository/repositories and accession number(s) can be found in the article/Supplementary Material.

## Ethics Statement

The animal study was reviewed and approved by the research board of the Ethics Committee of the Third Military Medical University under permit number AMUMEC-20201085.

## Author Contributions

JL, W-WH, and G-XQ performed the laboratory measurements. PJ, J-QL, W-HH, and Y-JJ made substantial contributions to the conception and design. X-RL, YX, JL, and QS participated in the experimental design and data analysis. JL drafted the manuscript. All authors read and approved the final manuscript.

## Conflict of Interest

The authors declare that the research was conducted in the absence of any commercial or financial relationships that could be construed as a potential conflict of interest.

## Publisher’s Note

All claims expressed in this article are solely those of the authors and do not necessarily represent those of their affiliated organizations, or those of the publisher, the editors and the reviewers. Any product that may be evaluated in this article, or claim that may be made by its manufacturer, is not guaranteed or endorsed by the publisher.

## Supplementary Material

Supplementary Figure 1 Detection of *vgrG2* gene in BPM, mutant and complemented strains.

Supplementary Figure 2 Growth characteristics of BPM, mutant and complemented strains.

Supplementary Table 1 The antibiotic susceptibility testing results of BPM, mutant and complemented strains.

## Abbreviations

BPM: *Burkholderia thailandensis* BPM
*B. pseudomallei*: *Burkholderia pseudomallei*
*E. coli*: *Escherichia coli*
*P. aeruginosa*: *Pseudomonas aeruginosa*.
