# Supplementary figures and images for: Characterization of a Type VI Secretion System vgrG2 Gene in the Pathogenicity of Burkholderia thailandensis BPM
Source: Front Microbiol. 2022 Jan 5;12:811343. doi: 10.3389/fmicb.2021.811343 (PMC8767068; doi:10.3389/fmicb.2021.811343)

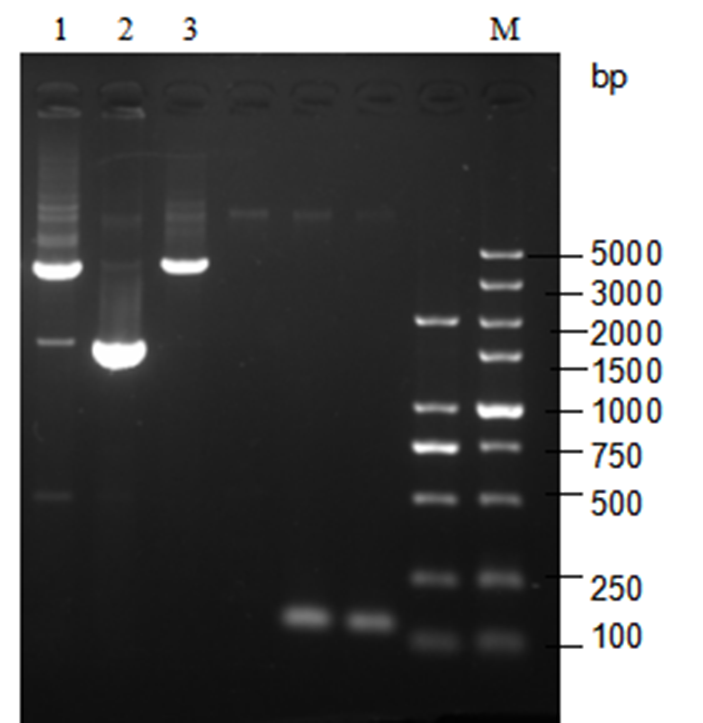

Supplement: Supplementary Figure 1 — Detection of vgrG2 gene in BPM, mutant and complemented strains. [file Image_1.tif]

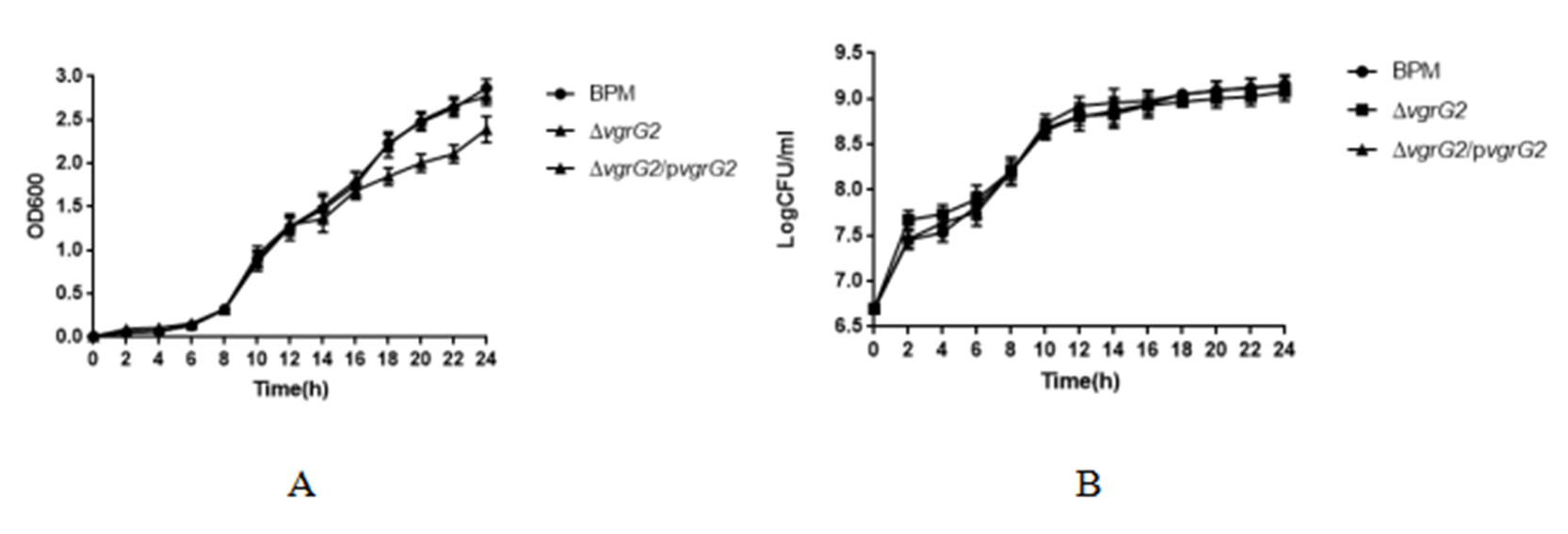

Supplement: Supplementary Figure 2 — Growth characteristics of BPM, mutant and complemented strains. [file Image_2.tif]
