# Supplementary material for: Characterization of a Type VI Secretion System vgrG2 Gene in the Pathogenicity of Burkholderia thailandensis BPM
Source: Front Microbiol. 2022 Jan 5;12:811343. doi: 10.3389/fmicb.2021.811343 (PMC8767068; doi:10.3389/fmicb.2021.811343)
Supplement: Supplementary Table 1 — The antibiotic susceptibility testing results of BPM, mutant and complemented strains. [file Table_1.docx]

**Supplementary Table 1. The antibiotic susceptibility testing results of BPM, mutant and complemented strains**

| Antimicrobial | MIC | Interpretation |
| --- | --- | --- |
| Amoxicillin/clavulanate | <= 4 | S |
| Ceftazidime | 2 | S |
| Imipenem | <= 1 | S |
| Tetracycline | 4 | S |
| Doxycycline | <= 0.5 | S |
| Trimethoprim/Sulfamethoxazole | 40 | S |
